# Supplementary material for: Zinc-enhanced activity of an antimicrobial halogenated phenazine against Streptococcus mutans and other gram-positive bacteria
Source: mSphere. 2026 Jan 20;11(2):e00585-25. doi: 10.1128/msphere.00585-25 (PMC12931261; doi:10.1128/msphere.00585-25)
Supplement: Supplemental material — Supplemental tables and figure. [file msphere.00585-25-s0001.pdf]

Table S1. Summary of final optical density values during MIC testing for HP-29 with or without metal supplementation.

**Media Supplementation**	***S. mutans* UA159**		***S. mutans ΔzccE***		***S. sanguinis* SK36**		***S. gordonii* DL-1**		***E. faecalis* OG1RF**		***S. aureus* RN4220**	
	**OD_{600}[a] (SD)[b]**	***p*-value[c]**	**OD_{600}[a] (SD)[b]**	***p*-value[c]**	**OD_{600}[a] (SD)[b]**	***p*-value[c]**	**OD_{600}[a] (SD)[b]**	***p*-value[c]**	**OD_{600}[a] (SD)[b]**	***p*-value[c]**	**OD_{600}[a] (SD)[b]**	***p*-value[c]**
BHI	0.733 (0.025)		0.657 (0.013)		0.619 (0.023)		0.516 (0.039)		0.850 (0.032)		1.123 (0.020)	
HP-29 only	0.125 (0.033)	<0.0001	0.167 (0.017)	0.0017	0.106 (0.004)	0.0004	0.146 (0.004)	0.0035	0.087 (0.004)	0.0005	0.076 (0.003)	<0.0001
BHI+ 1 mM Iron	0.674 (0.036)											
HP-29 + 1 mM Iron	0.220 (0.052)	0.0005										
BHI + 50 µM Manganese	0.705 (0.004)		0.691 (0.027)		0.626 (0.059)		0.573 (0.016)		0.841 (0.014)		1.370 (0.090)	
HP-29 + 50 µM Manganese	0.072 (0.002)	<0.0001	0.087 (0.014)	<0.0001	0.124 (0.015)	0.0029	0.116 (0.004)	<0.0001	0.080 (0.003)	<0.0001	0.067 (0.002)	0.0016
BHI + 250 µM Cobalt	0.567 (0.05)											
HP-29 + 250 µM Cobalt	0.117 (0.001)	0.0040										

BHI+ 500 µM Nickel	0.512											
	(0.058)											
HP-29 + 500 µM Nickel	0.161	0.0042										
	(0.077)											
BHI+ 10 µM Zinc	0.694		0.700									
	(0.021)		(0.061)									
HP-29 + 10 µM Zinc	0.136	0.0008	0.132	0.0037								
	(0.051)		(0.003)									
BHI+ 50 µM Zinc	0.719		0.679									
	(0.030)		(0.038)									
HP-29 + 50 µM Zinc	0.089	<0.0001	0.146	0.0012								
	(0.011)		(0.008)									
BHI+ 100 µM Zinc	0.709		0.572		0.577		0.550		0.878			
	(0.008)		(0.016)		(0.023)		(0.029)		(0.045)			
HP-29 + 100 µM Zinc	0.071	<0.0001	0.110	0.0001	0.095	0.0004	0.113	<0.0001	0.096	0.0011		
	(0.010)		(0.005)		(0.006)		(0.005)		(0.001)			
BHI+ 500 µM Zinc	0.724										1.167	
	(0.012)										(0.036)	
HP-29 + 500 µM Zinc	0.078	<0.0001									0.093	<0.0001
	(0.025)										(0.027)	

[a] OD_{600} values were obtained after 24 hrs of growth.

[b] SD: Standard deviation of at least three experiments.

[C] *p*-values indicate the significance obtained from Student's *t*-test, comparing the final OD_{600} values of each condition with those of the respective control medium lacking HP-29

Table S2. *S. mutans* differentially expressed genes when grown in BHI with 0.025μM HP-29 as compared to BHI medium.

Locus	Gene	Description	Fold change, Log2	*p*-value
SMU_182	*sloA*	metal ABC transporter ATP-binding protein	2.2507	4.04E-05
SMU_183	*sloB*	metal ABC transporter permease	2.6239	2.93E-05
SMU_184	*sloC*	metal ABC transporter substrate-binding protein	2.6482	4.23E-05
SMU_186	*sloR*	metal-dependent transcriptional regulator SloR	2.3081	1.84E-05
SMU_236c		TetR/AcrR family transcriptional regulator	3.6597	5.57E-07
SMU_237c		ABC transporter permease	5.5329	1.84E-07
SMU_238c		ABC transporter ATP-binding protein	5.342	6.99E-08
SMU_540	*dpr*	DNA starvation/stationary phase protection protein	-1.017	2.05E-03
SMU_635		VIT family protein	-1.2144	6.92E-07
SMU_768c		hypothetical protein	1.2317	1.26E-04
SMU_770c	*mntH*	Nramp family divalent metal transporter	1.194	3.47E-05
SMU_2057c	*zccE*	heavy metal translocating P-type ATPase	2.7787	1.38E-05

Table S3 *S. mutans* differentially expressed genes when grown in BHI with 0.025 µM HP-29 and 0.5 mM Zinc as compared to BHI medium.

Locus	Gene	Description	Fold change, Log2	*p*-value	Observed in zinc exposure study? (1)
		Upregulated genes			
SMU_05		DUF951 domain-containing protein	1.4703	2.83E-07	Yes
SMU_143c	def	peptide deformylase	1.1577	3.65E-06	
SMU_144c		cyclic nucleotide-binding domain-containing protein	1.5123	2.79E-06	
SMU_145		MFS transporter	1.2601	1.33E-04	
SMU_236c		TetR/AcrR family transcriptional regulator	1.7302	1.02E-04	
SMU_237c		ABC transporter permease	3.3266	4.76E-06	
SMU_238c		ABC transporter ATP-binding protein	3.4473	1.23E-06	
SMU_239c		VanZ family protein	1.4490	4.87E-05	Yes
SMU_363		MerR family transcriptional regulator	2.1643	2.20E-05	Yes
SMU_364	glnA	type I glutamate--ammonia ligase	2.3602	4.90E-05	Yes
SMU_365	gltB	glutamate synthase large subunit	1.1874	2.51E-03	Yes
SMU_383c		SDR family oxidoreductase	1.1039	2.25E-04	
SMU_424	copY	CopY/TcrY family copper transport repressor	1.6905	1.18E-06	Yes
SMU_426	copA	heavy metal translocating P-type ATPase	1.8385	6.15E-07	Yes
SMU_427	copZ	copper chaperone CopZ	2.2749	2.70E-08	Yes
SMU_503c		hypothetical protein	1.7932	1.19E-05	Yes
SMU_561c		NUDIX hydrolase	1.3835	2.03E-04	Yes
SMU_602		bile acid:sodium symporter family protein	1.2935	1.22E-02	Yes
SMU_609		SH3 domain-containing protein	1.5204	3.92E-06	Yes
SMU_610	spaP	cell surface antigen I/II	1.8388	4.52E-06	
SMU_629	sodA	superoxide dismutase SodA	1.0397	2.69E-04	Yes
SMU_647		O-methyltransferase	1.1339	3.31E-05	Yes

SMU_670	acnA	aconitate hydratase AcnA	5.1800	2.62E-09	Yes
SMU_671		citrate synthase	4.9365	2.00E-09	Yes
SMU_672	icd	NADP-dependent isocitrate dehydrogenase	4.7856	3.47E-09	Yes
SMU_673		putative ABC transporter permease	3.5720	3.06E-09	Yes
SMU_724		glycerophosphodiester phosphodiesterase	1.0352	8.76E-06	Yes
SMU_727		MerR family transcriptional regulator	1.5508	3.82E-07	Yes
SMU_728		NADP-dependent oxidoreductase	1.6433	6.86E-08	Yes
SMU_730		AbrB/MazE/SpoVT family DNA-binding domain-containing protein	1.5810	4.05E-04	
SMU_731		ABC transporter ATP-binding protein	1.0354	4.96E-03	
SMU_764	ahpC	alkyl hydroperoxide reductase subunit C	1.4888	1.33E-05	Yes
SMU_765	ahpF	alkyl hydroperoxide reductase subunit F	1.3951	7.23E-05	Yes
SMU_805c		amino acid ABC transporter ATP-binding protein	1.3623	3.40E-05	Yes
SMU_806c		ABC transporter substrate-binding protein/permease	1.4792	9.54E-06	Yes
SMU_838	gorA	glutathione-disulfide reductase	1.5155	4.87E-05	Yes
SMU_839		folylpolyglutamate synthase/dihydrofolate synthase family protein	1.0469	2.14E-07	Yes
SMU_911c		DUF308 domain-containing protein	1.7936	1.27E-06	Yes
SMU_913	gdhA	NADP-specific glutamate dehydrogenase	1.2891	2.84E-05	Yes
SMU_924	tpx	thiol peroxidase	1.3705	3.66E-04	Yes
SMU_925		hypothetical protein	1.4413	4.28E-05	Yes
SMU_984		CHAP domain-containing protein	1.8744	4.06E-06	Yes
SMU_991		ATP cone domain-containing protein	1.3025	4.18E-04	Yes
SMU_995		ABC transporter permease; iron chelate uptake ABC transporter family permease subunit	1.5195	1.80E-09	Yes
SMU_996		iron chelate uptake ABC transporter family permease subunit	1.6021	1.21E-08	Yes
SMU_997		ABC transporter ATP-binding protein	1.6657	5.75E-09	Yes
SMU_998		siderophore ABC transporter substrate-binding protein	1.8776	1.95E-09	Yes
SMU_999		hypothetical protein	1.3852	3.98E-05	Yes

SMU_1009		sensor histidine kinase	1.0455	2.36E-06	
SMU_1036		ABC transporter permease	1.0943	1.65E-04	
SMU_1037c		sensor histidine kinase KdpD	1.1970	8.89E-08	
SMU_1038c		response regulator transcription factor	1.1486	3.40E-07	Yes
SMU_1039c		glycosyltransferase family 8 protein	1.0738	3.29E-07	Yes
SMU_1040c		SDR family oxidoreductase	1.0486	3.29E-07	Yes
SMU_1048		CYTH domain-containing protein	1.1296	9.65E-06	Yes
SMU_1071c		alpha/beta hydrolase	1.1475	7.16E-05	
SMU_1072c		GNAT family N-acetyltransferase	2.9509	3.98E-05	
SMU_1073		formate--tetrahydrofolate ligase	1.1972	3.80E-05	
SMU_1175		sodium:alanine symporter family protein	2.0142	1.89E-02	Yes
SMU_1259		Eco57I restriction-modification methylase domain-containing protein	1.0866	1.01E-03	
SMU_1287		MerR family transcriptional regulator	1.4986	3.00E-04	Yes
SMU_1294		flavodoxin	1.0830	2.85E-06	
SMU_1296	yghU	glutathione-dependent disulfide-bond oxidoreductase	1.8087	6.14E-05	Yes
SMU_1297		bifunctional oligoribonuclease/PAP phosphatase NrnA	1.3242	1.32E-04	Yes
SMU_1396		glucan-binding protein; GbpC/Spa domain-containing protein	1.4807	2.83E-05	Yes
SMU_1451	budA	acetolactate decarboxylase	1.1546	7.13E-05	
SMU_1488c		DUF3884 family protein	1.2860	1.20E-02	Yes
SMU_1489		aldose 1-epimerase family protein	1.4538	5.50E-03	Yes
SMU_1490	lacG	6-phospho-beta-galactosidase	1.4547	5.47E-03	Yes
SMU_1491		lactose-specific PTS transporter subunit EIIC	1.2750	1.44E-02	Yes
SMU_1492	lacF	PTS lactose transporter subunit IIA	1.7617	6.67E-03	Yes
SMU_1493	lacD	tagatose-bisphosphate aldolase	1.4625	1.11E-02	Yes
SMU_1494	lacC	tagatose-6-phosphate kinase	1.3152	3.05E-02	Yes
SMU_1495	lacB	galactose-6-phosphate isomerase subunit LacB	1.4203	2.04E-02	Yes
SMU_1496	lacA	galactose-6-phosphate isomerase subunit LacA	1.0919	9.07E-02	Yes
SMU_1498	lacR	transcriptional regulator LacR	1.6593	2.00E-04	Yes

SMU_1519		amino acid ABC transporter ATP-binding protein	1.8444	3.76E-04	
SMU_1520		transporter substrate-binding domain-containing protein	2.0043	1.17E-04	
SMU_1521		amino acid ABC transporter permease	1.9538	4.53E-04	
SMU_1522		amino acid ABC transporter permease	1.8533	1.62E-04	
SMU_1602		NAD(P)H-dependent oxidoreductase	1.4341	5.75E-07	Yes
SMU_1603	gloA	lactoylglutathione lyase; VOC family protein	1.7343	5.35E-08	Yes
SMU_1649		exodeoxyribonuclease III	1.0461	1.47E-04	
SMU_1657c		P-II family nitrogen regulator	4.8278	2.61E-09	Yes
SMU_1658		ammonium transporter	4.7830	1.75E-08	Yes
SMU_1670c		YlbG family protein	1.0306	1.51E-04	Yes
SMU_1671c		YlbF family regulator	1.0970	9.29E-05	Yes
SMU_1682c		type 1 glutamine amidotransferase family protein	1.2879	3.00E-05	Yes
SMU_1700c	cidB	antiholin-like protein; LrgB family protein	1.3588	8.31E-04	
SMU_1704		PadR family transcriptional regulator	1.7332	8.63E-07	Yes
SMU_1705		DUF1700 domain-containing protein	1.5861	2.20E-05	Yes
SMU_1706		DUF4097 family beta strand repeat-containing protein	1.4942	1.65E-06	Yes
SMU_1753c	cas2	CRISPR-associated endonuclease Cas2	1.0091	5.69E-06	Yes
SMU_1758c	cas4	CRISPR-associated protein Cas4	1.1722	1.00E-05	Yes
SMU_1760c	cas7c	type I-C CRISPR-associated protein Cas7/Csd2	1.3129	6.86E-06	Yes
SMU_1763c	cas5c	type I-C CRISPR-associated protein Cas5c	1.1557	8.06E-06	Yes
SMU_1764c		CRISPR-associated helicase/endonuclease Cas3	1.2491	1.83E-05	Yes
SMU_1803c		DUF4230 domain-containing protein	1.1008	4.28E-05	Yes
SMU_1862		hypothetical protein	1.2022	6.65E-05	
SMU_1865	mutY	A/G-specific adenine glycosylase	1.6414	7.22E-06	Yes
SMU_1867c		zinc-dependent alcohol dehydrogenase family protein	1.2015	3.07E-04	Yes
SMU_1869	trxA	thioredoxin	1.1212	2.20E-04	Yes
SMU_1883		DUF956 family protein	1.0473	1.86E-05	Yes
SMU_1916		sensor histidine kinase; GHKL domain-containing protein	1.3364	2.90E-05	

SMU_1917		response regulator transcription factor	1.2788	2.10E-04	
SMU_1945		polyphosphate polymerase domain-containing protein	1.2728	1.21E-06	
SMU_1946		DUF4956 domain-containing protein	1.1446	5.51E-06	
SMU_1954	groL	chaperonin GroEL	1.0024	1.51E-02	Yes
SMU_1955	groES	co-chaperone GroES	1.1675	4.34E-03	Yes
SMU_1980c	comGG	competence type IV pilus minor pilin ComGG	1.2304	1.51E-03	
SMU_1981c	comGF	competence type IV pilus minor pilin ComGF	1.1081	3.30E-02	
SMU_1982c	comGE	competence type IV pilus minor pilin ComGE	1.2829	2.27E-03	
SMU_1985	comGB	competence type IV pilus assembly protein ComGB; type II secretion system F family protein	1.1965	3.77E-04	
SMU_1987	comGA; tadA	competence type IV pilus ATPase ComGA; Flp pilus assembly complex ATPase component TadA	1.0231	9.28E-03	
SMU_1988c		DUF1033 family protein	1.3324	1.94E-04	Yes
SMU_2057c	zccE	heavy metal translocating P-type ATPase	3.2372	4.93E-06	Yes
SMU_2074	nrdD	anaerobic ribonucleoside-triphosphate reductase	1.2458	2.55E-05	Yes
SMU_2129c		metal-sulfur cluster assembly factor	1.3921	7.17E-05	Yes
SMU_2133c		YhgE/Pip domain-containing protein	2.1617	1.80E-03	
		Downregulated genes			
SMU_16		APC family permease	-1.2789	4.51E-07	
SMU_20	mreC	rod shape-determining protein MreC	-1.4076	9.33E-08	
SMU_21	mreD	rod shape-determining protein MreD	-1.7769	7.55E-05	Yes
SMU_22	pcsB	peptidoglycan hydrolase PcsB	-1.6127	3.15E-08	
SMU_78	fruA	fructan beta-fructosidase	-3.1433	1.92E-03	
SMU_79		glycoside hydrolase family 32 protein	-3.1201	1.92E-03	
SMU_89c		formate/nitrite transporter family protein	-1.3447	8.69E-04	
SMU_100		PTS sugar transporter subunit IIB	-1.3521	5.75E-04	Yes
SMU_101		PTS sugar transporter subunit IIC	-1.5436	4.66E-04	Yes
SMU_102		PTS system mannose/fructose/sorbose family transporter subunit IID	-1.7091	2.90E-04	Yes
SMU_103		PTS sugar transporter subunit IIA	-1.5615	9.68E-04	Yes
SMU_104		alpha-glucosidase	-1.9403	1.48E-04	Yes

SMU_105		LacI family DNA-binding transcriptional regulator	-1.2667	2.68E-04		
SMU_133c		MFS transporter	-1.1840	1.29E-05		
SMU_148	adhE	bifunctional acetaldehyde-CoA/alcohol dehydrogenase	-3.3022	5.89E-03		
SMU_179		NADPH-dependent FMN reductase	-2.1115	6.96E-04		
SMU_180		flavocytochrome c	-2.2152	2.68E-03		Yes
SMU_263		APC family permease	-2.0155	2.15E-02		Yes
SMU_264	aguA	agmatine deiminase	-1.7908	2.82E-02		
SMU_265	arcC	carbamate kinase	-1.7957	5.03E-02		
SMU_292		helix-turn-helix transcriptional regulator; AraC family	-1.0539	1.41E-06	Yes	
SMU_308		SDR family oxidoreductase	-1.0345	7.07E-05		
SMU_401c		GNAT family N-acetyltransferase	-1.0201	2.67E-04		
SMU_402	pflB	formate C-acetyltransferase	-2.2237	1.18E-03	Yes	
SMU_438c		2-hydroxyacyl-CoA dehydratase	-1.5518	5.53E-06		
SMU_496	cysK	cysteine synthase A	-1.4018	1.46E-03		
SMU_500	raiA	ribosome hibernation-promoting factor, HPF/YfiA family	-1.1445	1.40E-03		
SMU_527		dihydrodipicolinate reductase; Gfo/Idh/MocA family oxidoreductase	-1.2734	4.20E-05		
SMU_531		chorismate mutase	-1.5423	5.23E-07		
SMU_532	trpE	anthranilate synthase component I	-1.3353	1.45E-05	Yes	
SMU_533		aminodeoxychorismate/anthranilate synthase component II	-1.1659	6.80E-05	Yes	
SMU_534	trpD	anthranilate phosphoribosyltransferase	-1.2002	5.90E-05	Yes	
SMU_535	trpC	indole-3-glycerol phosphate synthase TrpC	-1.0106	2.47E-04	Yes	
SMU_539c		A24 family peptidase	-1.2907	5.65E-04		
SMU_541		YqgQ family protein	-1.4364	3.16E-05	Yes	
SMU_542		ROK family glucokinase	-1.3615	2.63E-04	Yes	
SMU_543		rhodanese-like domain-containing protein	-1.4015	1.48E-04	Yes	
SMU_574c	lrgB	antiholin-like protein LrgB	-1.9930	5.78E-03		
SMU_575c	lrgA	holin-like protein LrgA	-1.9591	1.28E-03		

SMU_576	lytR	two-component system response regulator LytR	-1.3124	2.19E-03	Yes
SMU_577	lytS	two-component system sensor histidine kinase LytS	-1.4386	5.22E-04	Yes
SMU_611		DEAD/DEAH box helicase	-1.2952	1.51E-05	
SMU_616		hypothetical protein	-1.6179	1.99E-05	
SMU_618		hypothetical protein	-1.7692	2.57E-06	
SMU_636	nagB	glucosamine-6-phosphate deaminase NagB	-1.0549	2.87E-02	
SMU_675	ptsP	phosphoenolpyruvate--protein phosphotransferase	-1.2075	1.12E-04	
SMU_697	infC	translation initiation factor IF-3	-1.0166	9.81E-07	
SMU_699	rplT	50S ribosomal protein L20	-1.1085	1.58E-06	
SMU_772		YSIRK-type signal peptide-containing protein	-1.1262	1.10E-04	
SMU_799c		thioesterase family protein	-1.3430	1.96E-03	
SMU_803c		ATP-binding cassette domain-containing protein	-1.3958	1.54E-06	
SMU_832		hypothetical protein	-1.0241	1.44E-06	Yes
SMU_865	rpsP	30S ribosomal protein S16	-1.3165	3.15E-08	
SMU_866		KH domain-containing protein	-1.0604	5.83E-07	
SMU_876		AraC family transcriptional regulator	-1.0752	4.43E-03	
SMU_877		alpha-galactosidase	-2.1691	2.87E-04	
SMU_878	msmE	sugar-binding protein MsmE	-2.3942	1.90E-04	Yes
SMU_879		carbohydrate ABC transporter permease	-2.3377	3.20E-04	Yes
SMU_880		carbohydrate ABC transporter permease	-2.4045	2.03E-04	Yes
SMU_881	gtfA	sucrose phosphorylase	-2.3388	9.65E-05	Yes
SMU_882		ABC transporter ATP-binding protein	-2.2131	1.99E-04	Yes
SMU_883	dexB	glucan 1,6-alpha-glucosidase DexB	-2.1114	1.75E-04	Yes
SMU_886		galactokinase	-1.1933	1.44E-04	
SMU_905		ABC transporter ATP-binding protein	-1.0735	3.22E-05	Yes
SMU_906		ABC transporter ATP-binding protein	-1.1979	2.92E-05	Yes
SMU_910	gtfD	glucosyltransferase-S	-2.9764	1.35E-09	Yes
SMU_937	mvaD	diphosphomevalonate decarboxylase	-1.0642	4.89E-04	
SMU_957	rplJ	50S ribosomal protein L10	-1.7127	2.90E-08	
SMU_960	rplL	50S ribosomal protein L7/L12	-1.6050	1.51E-07	

SMU_980		beta-glucoside-specific PTS transporter subunit IIABC	-2.4074	3.64E-05	Yes
SMU_992		hypothetical protein	-1.6219	1.61E-06	Yes
SMU_1041		ABC transporter ATP-binding protein	-1.0846	2.80E-06	
SMU_1042		hypothetical protein	-1.1468	1.18E-06	
SMU_1062		ABC transporter permease/substrate binding protein	-1.7049	2.89E-05	Yes
SMU_1063		glycine betaine/L-proline ABC transporter ATP-binding protein	-1.5922	2.19E-05	Yes
SMU_1070c		LytTR family DNA-binding domain-containing protein	-1.0413	3.04E-02	
SMU_1077		phospho-sugar mutase	-2.3240	3.01E-04	Yes
SMU_1091		putative cross-wall-targeting lipoprotein signal domain-containing protein	-1.7818	2.42E-09	Yes
SMU_1093		ABC transporter permease	-1.0788	3.14E-04	
SMU_1124		pyrimidine-nucleoside phosphorylase	-1.4986	2.78E-04	Yes
SMU_1125c		class I SAM-dependent methyltransferase	-1.6687	1.70E-04	Yes
SMU_1185		PTS mannitol transporter subunit IICB	-1.8002	4.75E-02	Yes
SMU_1284c		NAD(P)-dependent oxidoreductase	-1.1361	3.42E-05	
SMU_1286c		MFS transporter	-1.3188	2.50E-05	Yes
SMU_1302	adcA	zinc ABC transporter substrate-binding protein AdcA	-1.3449	3.34E-06	Yes
SMU_1334	mubP	mutanobactin A biosynthesis phosphopantetheinyl transferase MubP	-2.6908	2.27E-06	
SMU_1335c	mubJ	mutanobactin A biosynthesis reductase MubJ	-2.5377	2.62E-06	Yes
SMU_1336	mubI	mutanobactin A biosynthesis transacylase MubI	-2.7183	1.52E-06	Yes
SMU_1337c	mubM	mutanobactin A biosynthesis alpha/beta hydrolase MubM	-2.8711	1.54E-06	
SMU_1338c	mubZ	mutanobactin A system MFS transporter MubZ	-3.0089	5.45E-07	
SMU_1339	mubD	mutanobactin A non-ribosomal peptide synthetase MubD	-3.0523	8.76E-07	Yes

SMU_1340	mubC	mutanobactin A non-ribosomal peptide synthetase MubC	-3.0810	1.06E-06	Yes
SMU_1341c	mubB	mutanobactin A non-ribosomal peptide synthetase MubB	-3.0851	1.11E-06	Yes
SMU_1342	mubA	mutanobactin A non-ribosomal peptide synthetase MubA	-2.9289	3.92E-06	Yes
SMU_1343c	mubH	mutanobactin A polyketide synthase MubH	-2.6412	4.70E-06	Yes
SMU_1344c	mubG	mutanobactin A biosynthesis transacylase MubG	-2.6111	5.31E-06	Yes
SMU_1345c	mubE	mutanobactin A non-ribosomal peptide synthetase MubE	-2.3929	5.05E-06	Yes
SMU_1346	mubT	mutanobactin A biosynthesis thioesterase MubT	-2.1237	1.53E-05	Yes
SMU_1347c	mubY	mutanobactin A system ABC transporter permease subunit MubY	-1.4128	1.61E-04	Yes
SMU_1348c	mubX	mutanobactin A system ABC transporter ATP-binding subunit MubX	-1.1738	1.64E-04	Yes
SMU_1365c	mubY	mutanobactin A system ABC transporter permease subunit MubY	-1.3729	2.76E-04	Yes
SMU_1366c	mubX	mutanobactin A system ABC transporter ATP-binding subunit MubX	-1.1343	2.85E-04	Yes
SMU_1390		Pr6Pr family membrane protein	-1.6196	5.54E-07	
SMU_1410		FAD-dependent oxidoreductase	-2.4865	4.45E-04	
SMU_1411		MFS transporter	-2.4402	4.13E-04	
SMU_1424	lpdA	dihydrolipoyl dehydrogenase	-1.0765	2.47E-02	
SMU_1425		ATP-dependent Clp protease ATP-binding subunit; AAA family ATPase	-2.0085	6.02E-03	
SMU_1536	glgA	glycogen synthase GlgA	-1.3154	6.09E-02	Yes
SMU_1537	glgD	glucose-1-phosphate adenylyltransferase subunit GlgD	-1.4409	4.81E-02	Yes
SMU_1538		glucose-1-phosphate adenylyltransferase	-1.3459	5.60E-02	Yes
SMU_1564		glycogen/starch/alpha-glucan phosphorylase	-1.3555	3.20E-04	
SMU_1565	malQ	4-alpha-glucanotransferase	-1.1439	1.15E-04	
SMU_1568		extracellular solute-binding protein	-1.5707	3.00E-04	

SMU_1569		carbohydrate ABC transporter permease	-1.0878	3.39E-03	
SMU_1570		sugar ABC transporter permease	-1.2668	3.60E-04	
SMU_1571	ugpC	ABC transporter ATP-binding protein	-1.1911	7.46E-04	
SMU_1590		alpha-amylase	-1.4185	2.52E-04	Yes
SMU_1591	ccpA	catabolite control protein A	-1.4661	4.63E-04	Yes
SMU_1595		carbonic anhydrase	-1.3908	2.04E-08	
SMU_1734		acetyl-CoA carboxylase carboxyl transferase subunit alpha	-1.8238	1.25E-09	Yes
SMU_1735	accD	acetyl-CoA carboxylase, carboxyltransferase subunit beta	-2.1461	8.43E-09	Yes
SMU_1736	accC	acetyl-CoA carboxylase biotin carboxylase subunit	-2.0359	8.40E-10	Yes
SMU_1737	fabZ	3-hydroxyacyl-ACP dehydratase FabZ	-1.9017	1.10E-09	Yes
SMU_1738	accB	acetyl-CoA carboxylase biotin carboxyl carrier protein	-2.0349	2.29E-10	Yes
SMU_1739	fabF	beta-ketoacyl-ACP synthase II	-1.9858	1.22E-09	Yes
SMU_1740	fabG	3-oxoacyl-[acyl-carrier-protein] reductase	-1.9597	4.77E-10	Yes
SMU_1741	fabD	ACP S-malonyltransferase	-1.8476	1.00E-09	Yes
SMU_1742c	fabK	enoyl-[acyl-carrier-protein] reductase FabK	-1.7209	1.71E-09	Yes
SMU_1743		acyl carrier protein	-1.0489	1.01E-06	
SMU_1744		beta-ketoacyl-ACP synthase III	-1.8073	3.91E-08	
SMU_1745c		MarR family winged helix-turn-helix transcriptional regulator	-1.9359	1.32E-08	
SMU_1812		ISL3-like element ISSmu2 family transposase	-1.0243	1.14E-03	
SMU_1841		sucrose-specific PTS transporter subunit IIBC	-2.4634	3.16E-05	Yes
SMU_1843		sucrose-6-phosphate hydrolase	-1.6404	1.35E-04	Yes
SMU_1844		LacI family DNA-binding transcriptional regulator	-1.4908	5.83E-04	Yes
SMU_1856c		TraX family protein	-1.2719	4.10E-05	
SMU_1877	manX	PTS sugar transporter subunit IIB	-2.2329	1.62E-04	Yes
SMU_1878		PTS mannose/fructose/sorbose transporter subunit IIC	-2.0374	1.92E-04	Yes

SMU_1879		PTS system mannose/fructose/sorbose family transporter subunit IID	-2.0353	8.89E-05	Yes
SMU_1895c		Blp family class II bacteriocin	-1.5410	8.09E-06	
SMU_1924	gcrR	response regulator GcrR	-1.4267	7.48E-07	
SMU_1927		ABC transporter ATP-binding protein	-2.0206	1.72E-06	Yes
SMU_1928		ABC transporter permease; FtsX-like permease family protein	-2.5395	4.73E-07	Yes
SMU_1956c		hypothetical protein	-4.7765	2.03E-05	
SMU_1957		PTS system mannose/fructose/sorbose family transporter subunit IID	-5.0019	1.07E-05	
SMU_1958c		PTS sugar transporter subunit IIC	-5.0638	7.44E-06	
SMU_1960c		PTS sugar transporter subunit IIB	-5.4203	2.21E-06	
SMU_1961c		PTS sugar transporter subunit IIA	-5.1307	8.48E-06	
SMU_2000	rplQ	50S ribosomal protein L17	-1.6450	5.94E-10	
SMU_2001		DNA-directed RNA polymerase subunit alpha	-1.6056	2.24E-09	
SMU_2002	rpsK	30S ribosomal protein S11	-1.5641	1.15E-09	
SMU_2003	rpsM	30S ribosomal protein S13	-1.3464	8.25E-09	
SMU_2004	infA	translation initiation factor IF-1	-1.5022	9.25E-09	
SMU_2005		adenylate kinase	-1.6492	4.65E-10	
SMU_2006	secY	preprotein translocase subunit SecY	-1.4563	2.74E-08	
SMU_2007	rplO	50S ribosomal protein L15	-1.4907	2.45E-08	
SMU_2008	rpmD	50S ribosomal protein L30	-1.6160	4.96E-09	
SMU_2009	rpsE	30S ribosomal protein S5	-1.5092	8.02E-08	
SMU_2010	rplR	50S ribosomal protein L18	-1.6269	6.53E-09	
SMU_2011	rplF	50S ribosomal protein L6	-1.5695	9.15E-09	
SMU_2012	rpsH	30S ribosomal protein S8	-1.4847	1.31E-08	
SMU_2014		type Z 30S ribosomal protein S14	-1.5785	1.14E-08	
SMU_2015	rplE	50S ribosomal protein L5	-1.6522	3.76E-09	
SMU_2016	rplX	50S ribosomal protein L24	-1.5856	1.12E-08	
SMU_2017	rplN	50S ribosomal protein L14	-1.6980	1.75E-08	
SMU_2018	rpsQ	30S ribosomal protein S17	-1.6401	6.96E-09	
SMU_2019	rpmC	50S ribosomal protein L29	-1.8014	1.13E-08	

SMU_2020	rplP	50S ribosomal protein L16	-1.5926	2.61E-09	
SMU_2021	rpsC	30S ribosomal protein S3	-1.6299	9.14E-09	
SMU_2022	rplV	50S ribosomal protein L22	-1.6097	6.02E-09	
SMU_2023c	rpsS	30S ribosomal protein S19	-1.6463	4.84E-09	
SMU_2167	rplB	50S ribosomal protein L2	-1.6137	1.64E-08	
SMU_2166		50S ribosomal protein L23	-1.7209	4.32E-09	
SMU_2024c	rplD	50S ribosomal protein L4	-1.7590	9.77E-09	
SMU_2025	rplC	50S ribosomal protein L3	-1.7156	4.15E-09	
	rpsJ	30S ribosomal protein S10	-1.4787	2.20E-09	
SMU_2028		glycoside hydrolase family 68 protein	-2.9033	5.75E-06	Yes
SMU_2032	rpsB	30S ribosomal protein S2	-1.0659	1.01E-06	
SMU_2046c		endonuclease/exonuclease/phosphatase family protein	-1.4498	5.95E-05	
SMU_2047		PTS transporter subunit IIBC	-1.7082	3.50E-06	
SMU_2080	brsR	bacteriocin genes transcriptional regulator BrsR; LytTR family transcriptional regulator	-1.0768	2.39E-03	
SMU_2127		NAD-dependent succinate-semialdehyde dehydrogenase	-2.2005	2.25E-03	
SMU_2128	ilvD	dihydroxy-acid dehydratase	-1.1960	4.25E-05	

1. Ganguly T, Peterson AM, Burkholder M, Kajfasz JK, Abranches J, Lemos JA. 2022. ZccE is a Novel P-type ATPase That Protects Streptococcus mutans Against Zinc Intoxication. PLoS Pathog 18:e1010477.

Table S4. qRT-PCR primers used in this study.

Primer	Sequence (5' – 3')
zccERTFwd	CGCATATATTGATGGCGTTG
zccERTRev	TGTCAAACGTCTGGCTTCTG
copARTFwd	GGCAGTTGCTGGTGCAGG
copARTRev	GGGCAAGCCAACCATACTTCCC
sloCRTFwd	CAGTGAGCGATAGTGTTAAGAC
sloCRTRev	CTCTTGTTTTTAGGATCTTTTTCAGATAG
mntHRTFwd	CAATCAGAGCGTTCGTGTTCC
mntHRTRev	CGCCAACAACGCTGGTAATC
smu.237RTFwd	GCAAGGATTATAAAACTGAAAAAGC
smu.237RTRev	GCTTTTAATAGCTTGCCGC

Figure S1

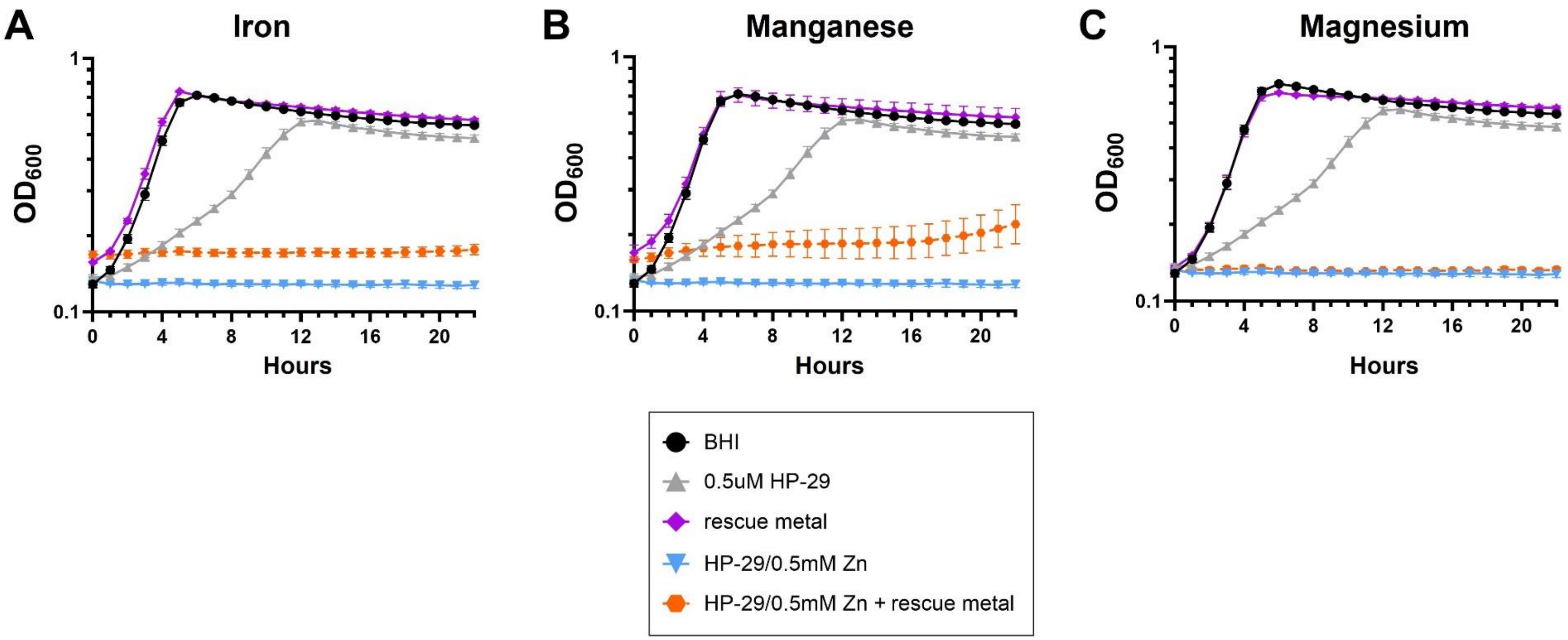

Fig. S1. Divalent metal cations cannot rescue the growth inhibition of *S. mutans* caused by exposure to HP-29 + zinc. *S. mutans* UA159 was grown in BHI media containing 0.5 μM HP-29 with or without the addition of 0.5mM zinc. The media was supplemented with the divalent metal cations (A) iron, 1 mM, (B) manganese, 1 mM, or (C) magnesium, 10 mM. Data represent averages and standard deviations of at least 3 independent experiments.
